# A novel anti-PSMA human scFv has the potential to be used as a diagnostic tool in prostate cancer

**DOI:** 10.18632/oncotarget.10697

**Published:** 2016-07-19

**Authors:** Donghui Han, Jieheng Wu, Yueheng Han, Ming Wei, Sen Han, Ruihe Lin, Ziyong Sun, Fa Yang, Dian Jiao, Pin Xie, Lingling Zhang, An-Gang Yang, Aizhi Zhao, Weihong Wen, Weijun Qin

**Affiliations:** ^1^ Department of Urology, Xijing Hospital, Fourth Military Medical University, 710032 Xi'an, China; ^2^ State Key Laboratory of Cancer Biology, Department of Immunology, Fourth Military Medical University, 710032 Xi'an, China; ^3^ Department of Research & Development, OriMAbs Ltd. Science Center, Philadelphia, PA, 19104, USA; ^4^ Department of Urology, Tangdu Hospital, Fourth Military Medical University, 710038 Xi'an, China; ^5^ Department of Research & Development, Hangzhou Immusource Biotechnology Company, Ltd., Hangzhou, 310010, China; ^6^ Institute of Molecular Medicine, Nanjing University, Nanjing, 210093, China

**Keywords:** prostate cancer, prostate specific membrane antigen, PSMA, single chain antibody fragment, diagnostic imaging

## Abstract

Prostate cancer (PCa) is the most commonly diagnosed malignancy and the second leading cause of cancer related death in men. The early diagnosis and treatment of PCa are still challenging due to the lack of efficient tumor targeting agents in traditional managements. Prostate specific membrane antigen (PSMA) is highly expressed in PCa, while only has limited expression in other organs, providing an ideal target for the diagnosis and therapy of PCa. The antibody library technique has opened the avenue for the discovery of novel antibodies to be used in the diagnosis and therapy of cancer. In this paper, by screening a large yeast display naive human single chain antibody fragment (scFv) library, we obtained a high affinity scFv targeting PSMA, called gy1. The gy1 scFv was expressed in *E.coli* and purified via a C terminal 6His tag. The binding affinity of gy1 was shown to be at the nanomolar level and gy1 can specifically bind with PSMA positive cancer cells, and binding triggers its rapid internalization through the endosome-lysosome pathway. The specific targeting of gy1 to PSMA positive tumor tissues was also evaluated *in vivo*. We showed that the IRDye800CW labeled gy1 can efficiently target and specifically distribute in PSMA positive tumor tissues after being injected into xenograft nude mice. This study indicated that the novel antibody gy1 could be used as a great tool for the development of PSMA targeted imaging and therapy agents for PCa.

## INTRODUCTION

Prostate cancer (PCa) is the most common malignancy and the second leading cause of cancer death in men, which causes 913,000 new cases and over 261,000 deaths worldwide each year [1]. In early diagnosed PCa that is restricted in the prostate gland, the five year survival rate is nearly 100%.Therefore, early and accurate diagnosis is crucial for the better outcome of PCa patients. Traditional imaging techniques for tumor diagnosis, such as MRI, CT, and bone scanning, are based on the anatomical differences between tumor tissues and normal tissues, and the sensitivity and specificity is not ideal [2, 3]. Antibody based molecular imaging is bringing revolutionary improvement for early tumor diagnosis. It enables not only sensitivity, but also specific detection of certain tumors even at their occult stage. Thus, discovery of novel antibodies is crucial for the diagnostic tumor imaging.

The prostate specific membrane antigen (PSMA) is abundantly expressed in PCa, especially in poorly differentiated, metastatic, and hormone-refractory cases, while only has limited expression in other organs. This expression pattern guarantees PSMA being an ideal target for the diagnosis and therapy of PCa [4–8]. Besides, PSMA has also been found to be highly expressed in solid tumor vasculature, but not in normal vascular endothelium, indicating that the PSMA targeted tools may have even wider application [9, 10]. Like other tumor antigens, PSMA has also been exploited in the discovery of specific antibodies. But currently all of the PSMA targeting antibodies under development are full antibodies, and most of them are murine monoclonal antibodies. The murine origin, slow clearance rate and poor tumor penetration of these full antibodies severely limited their application in tumor imaging and therapy. Smaller antibodies have more advantages for targeted imaging and therapy because they have faster clearance rate and deeper tissue penetration capability. Therefore, the discovery of novel smaller PSMA specific human antibodies are needed for the development of specific agents for PCa imaging and therapy. Single chain antibody fragment (scFv) is a kind of small engineered antibody, in which the variable regions of heavy chain (V_H_) and light chain (V_L_) are joined together by a flexible polypeptide linker. There have several different ways to get scFvs. The antibody library technique provides an ideal method for the discovery of novel antibodies. Antibody library technique has the advantage of direct isolation of fully human antibodies, which is preferred to be used in clinic due to the neglectable immunogenicity. However, the library size is in direct proportion to the affinity of the antibodies isolated. Therefore, a large size of the antibody library is the prerequisite for successful isolation of high affinity antibodies.

In our previous study, we have constructed a 1×10^11^ yeast-display naive human scFv library from peripheral blood mononuclear cells that were isolated from 5 liter peripheral blood from 56 healthy donors, providing the possibility to obtain high affinity PSMA specific scFv. In this study, we panned the library using human PSMA extracellular domain and obtained a specific scFv named gy1. To evaluate the potential application of gy1 for imaging and therapy of PCa, gy1 scFv was expressed and purified in *E. coli*, and the binding affinity and specificity was evaluated. Our results showed that gy1 can specifically bind with PSMA positive cancer cells, and the binding triggers its significant internalization through the endosome-lysosome pathway. Furthermore, after being injected into xenograft nude mice, IRDye800CW labeled gy1 can specifically and efficiently target PSMA positive tumor *in vivo*. Our results suggest that gy1 deserves further evaluation to be used in diagnostic imaging and targeted therapy of PCa.

## RESULTS

### Isolation and identification of gy1 scFv from a yeast-display human scFv library

The yeast-display naive scFv library was panned against recombinant human PSMA extracellular domain sequentially by magnetic and flow sorting as described previously [11]. Briefly, the PSMA binding yeast population was magnetically enriched using progressively decreased concentrations of PSMA protein. Yeast population with high affinity to PSMA was further isolated using flow cytometry and the top 0.1% high affinity population was used and converted into secretory scFv expression and individual clones were identified subsequently. Among the 384 clones evaluated, 260 had positive antigen binding signals (more than two folds of the blank control), from which 96 clones with highest signals were selected for further verification using recombinant Fc as control antigen. It turned out that all the 96 clones are PSMA specific, among which, 30 clones were sequenced and turned out to be the same scFv except several spot mutations among them. The scFv that has highest ELISA signal was named gy1 and used for further study.

### Expression, purification of gy1 in yeast and affinity measurement by capture ELISA

The YVH10 clone that contains gy1 scFv gene was expanded and induced to express gy1 protein. Secretory gy1 was purified from the yeast supernatant using Nickel resin since a 6His tag was designed at the C-terminus (Supplementary Figure 1A). Capture ELISA was applied to measure the affinity of gy1, anti-Flag antibody was coated on plates to avoid the potential distort of the structure of the coating antigen. Diluted purified gy1 protein were then added and followed by incubation of biotinylated PSMA and Streptavidin-HRP sequentially, and colorimetric signals were developed with TMB substrate solution. Result showed that the affinity of the yeast expressed gy1 is pretty high (Kd = 1.2 nM)(Supplementary Figure 1B).

### Expression, purification of gy1 in *E.coli*

**Figure 1:**
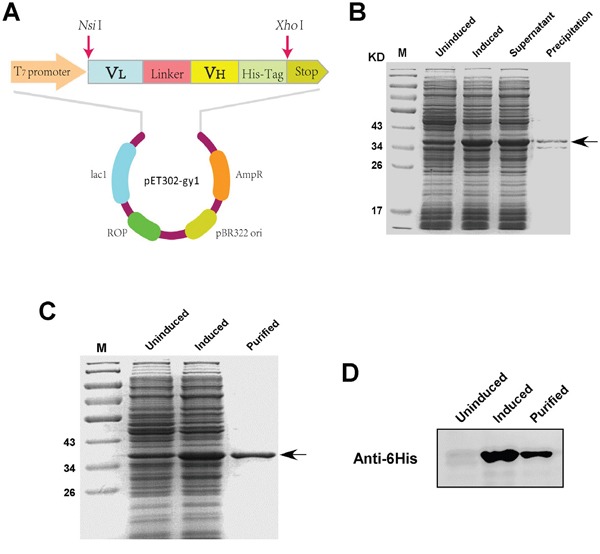
Expression and purification of gy1 in *E.coli* **A.** Schematic diagram of gy1 expressing plasmid. **B.** SDS-PAGE to show the induced and soluble gy1 expression. **C.** SDS-PAGE to show the purified gy1 protein. **D.** Western blot to identify the purified gy1 protein.

To establish a better way for gy1 production with lower cost, we pursued to express and purify gy1 in prokaryotic expression system. Gy1 gene was cloned into prokaryotic expressing vector pET302 (named pET302-gy1) (Figure 1A). The pET302-gy1 plasmid was then transformed into *E.coli* BL21 for inductive expression. IPTG concentration, induction time and temperature were optimized. Maximal soluble gy1 expression condition was determined, which was 0.05 mM IPTG induction at 30^°^C. The molecular weight of gy1 was found to be around 37kDa after being separated by 12% SDS-PAGE, which is consistent with prediction (Figure 1B). The gy1 protein was then purified by affinity chromatography using Ni^2+^-NTA column and the purified gy1 protein was further confirmed by Western blot using anti-6His antibody (Figure 1C, 1D). After calculation, we found that the production of gy1 in *E. coli* is about 7.5 mg/L.

### Gy1 can specifically bind PSMA positive cancer cells

**Figure 2:**
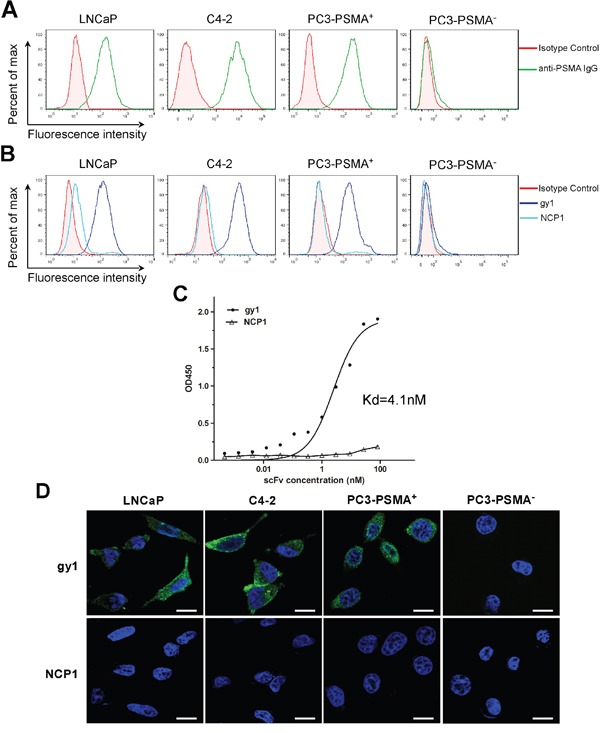
Gy1 can specifically bind and internalize into PSMA positive cancer cells **A.** Flow cytometry analysis to show the PSMA expression on different prostate cancer cells. **B.** Flow cytometry analysis to show the binding of gy1 to PSMA positive cancer cells. LNCaP, C4-2, PC3-PSMA^+^and PC3-PSMA^−^ cells were incubated with 100 nM of gy1 and followed by FITC-conjugated secondary antibody. NCP1 was used as negative control. **C.** Cellular ELISA to show the binding affinity of gy1. The Kd was calculated using non-linear regression analysis of a one-site binding hyperbola equation of GraphPad Prism 5.0 software. Representative result was shown from 3 independent experiments. **D.** Immunofluorescence staining to show the internalization of gy1 into PSMA positive cancer cells. Gy1 was incubated with LNCaP, C4-2, PC3-PSMA^+^ and PC3-PSMA^−^ cells for 2 h before immunofluorescence staining. Scale bar = 25 μm.

To evaluate the binding ability of gy1 with membrane expressed PSMA, we first examined the expression of PSMA in different prostate cancer cells, i.e., LNCaP, C4-2 and PC3 by flow cytometry. As shown in Figure 2A, LNCaP and C4-2 cells have positive PSMA expression, while PC3 cell has negative PSMA expression, which is consistent with previous reports [12]. The PSMA negative PC3 cells were infected with PSMA expressing lentivirus and luciferase expressing lentivirus to obtain the PC3-PSMA^+^ cell, and PC3 cells infected only with luciferase expressing lentivirus was named PC3-PSMA^−^ cell. Both cells were used for the *in vivo* study, since the LNCaP cell is hard to form xenograft in nude mice. The result of flow cytometry showed that PSMA expression can be detected in PC3-PSMA^+^ cells, indicating the stable PSMA-expressing PC3 cells were successfully established. Gy1 scFv was then evaluated to find out whether it can specifically bind with PSMA positive cancer cells. The four kinds of cells were incubated with purified gy1 followed by FITC-conjugated anti-6His antibody incubation, and were analyzed by flow cytometry. Results showed that gy1 can bind all three PSMA positive cells, but not the PSMA negative PC3-PSMA^−^ cells (Figure 2B). These result indicate that gy1 can specifically bind PSMA positive cancer cells.

Cellular ELISA was used to measure the affinity of *E.coli* expressed gy1. PSMA-positive C4-2 cells were incubated with different concentrations of gy1 or a control scFv NCP1, an anti-HER2 scFv, followed by incubation with HRP-conjugated anti-6His antibody and chromogenic reaction. Results showed that gy1 can bind PSMA-positive C4-2 cells at a high affinity of Kd = 4.1 nM (Figure 2C).

### Binding of gy1 with membrane PSMA triggers its instant internalization

Antibody internalization is necessary for an antibody to deliver toxic drugs or other payloads into target cells. To investigate the internalization capability of gy1, gy1 were incubated with the four cell lines for 2 h at 37^°^C before immunofluorescent staining. The control NCP1, an anti-HER2 scFv, was used as a negative control. Results showed that strong fluorescence signal can be observed in the cytoplasm of PSMA positive LNCaP, C4-2 and PC3-PSMA^+^ cells. While in PSMA negative PC3-PSMA^−^ cells, no fluorescence signal can be detected (Figure 2D). These results demonstrated that gy1 can effectively internalize into PSMA positive cells.

### Gy1 co-localizes with endosome and lysosome, but not Golgi or ER

**Figure 3:**
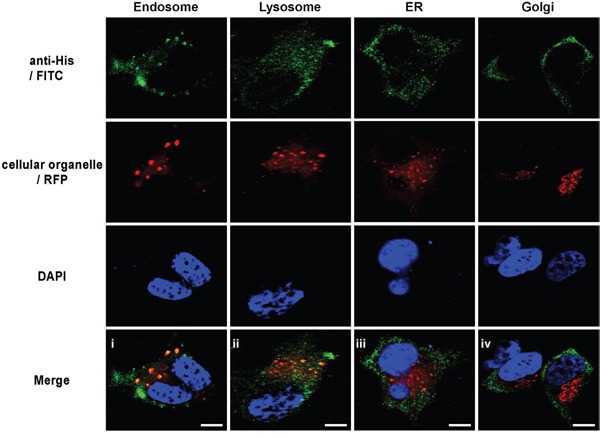
Gy1 co-localizes with endosome and lysosome, but not Golgi or ER Immunofluorescent staining to show the co-localization of gy1 with different cellular organelle marker in C4-2 cells. Cells were incubated with 200 nM gy1 for 4 h before immunofluorescent staining by anti-6His IgG and FITC-conjugated secondary antibody, different cellular organelle markers (RFP-labeled) and nucleus (blue) were co-stained. Scale bar = 10 μm.

To investigate the subcellular transportation of gy1 after internalization, immunofluorescent staining was performed to examine the co-localization of gy1 (green fluorescence) with certain cellular organelles, including endosome, lysosome, Golgi and ER (red fluorescence) in C4-2 cells. Results showed that after 4 h incubation, gy1 was predominantly accumulated in endosomes and lysosomes (yellow fluorescence, Figure 3), suggesting that gy1 internalizes into target cells through the endosome-lysosome pathway. No overlap signal could be found between the signals of gy1 and Golgi or ER.

Internalized protein mainly have two trafficking pathways. One is directly through endosome to lysosome and the other is from Golgi apparatus to ER, which is called retrograde trafficking and is commonly used for recycling transportation [13]. To avoid the possibility that we might miss the best time point to observe the overlap between gy1 and Golgi or ER marker, we further incubate gy1 with C4-2 cell for different time points. Results showed that even being incubated for different time points, still no co-localization could be observed between gy1 and Golgi or ER (Supplementary Figure 2), which abrogates the possibility of the second trafficking pathway after gy1internalization.

### Gy1 specifically enriches in PSMA positive tumor in xenograft mouse model

**Figure 4:**
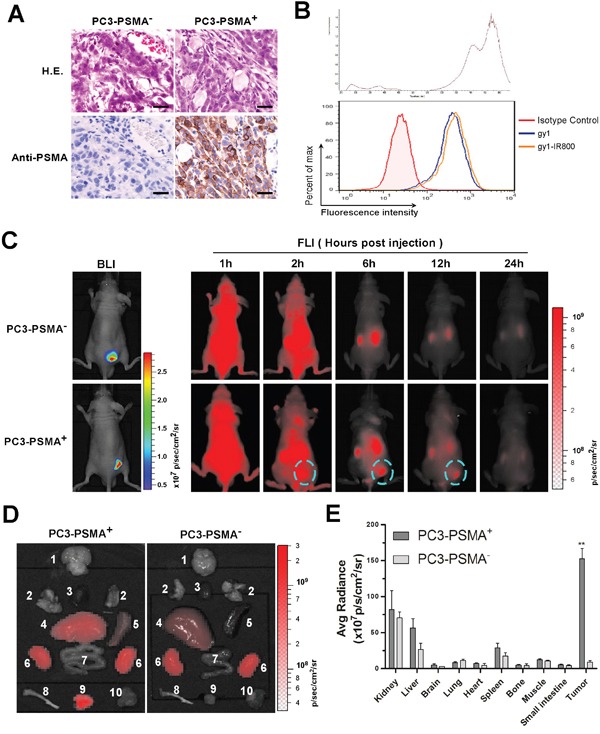
Gy1 specifically enriches in PSMA positive tumor in xenograft mouse model **A.** Validation of a PCa xenograft nude mouse model. Two weeks after inoculation, PCa tumor tissues were isolated and identified by H&E staining and immunohistochemistry staining using anti-PSMA antibody. **B.** Flow cytometry analysis to show IRDye800CW-labeled gy1 maintained the ability to bind PC3-PSMA^+^ cells. **C.** The dynamic distribution of IRDye800CW-labeled gy1 in PC3-PSMA^+^ or PC3-PSMA^−^ xenograft nude mice. Bioluminescence imaging (BLI) was acquired to identify the PCa tumor tissues. The distribution of IRDye800CW-labeled gy1 was monitored at indicated time points in the same mouse of each group. Representative result was shown. **D.** Bio-distribution of IRDye800CW-labeled gy1 in different organs at 12 h after intravenous injection. Mice were sacrificed at 12 h after gy1 injection and different tissues were isolated for bio-distribution evaluation. 1, brain; 2, lung; 3, heart; 4, liver; 5, spleen; 6, kidney; 7, small intestine; 8, bone; 9, PC3-PSMA^+^ or PC3-PSMA^−^ tumor tissue; and 10, muscle. **E.** Fluorescence quantification of different organs in D. The signal intensity of the tumor tissues and different organs was measured by IVIS software. Bar, mean values; Error bar, SD; n =5; ^**^P < 0.001 versus PC3-PSMA^−^ tissue.

The capability and efficiency of gy1 for PSMA targeting *in vivo* was evaluate using PSMA positive and negative xenograft nude mice. The PC3-PSMA^+^ and PC3-PSMA^−^ cells were subcutaneously injected on back near the right thigh of nude mice, two weeks after inoculation, tumor tissue was isolated and H&E and immunohistochemistry staining were performed to examine the tissue morphology and PSMA expression. Results showed that the PC3-PSMA^+^ prostate cancer tissues showed positive PSMA staining, while no PSMA expression can be found in the PC3-PSMA^−^ prostate cancer tissues (Figure 4A). Tumor formation and location was also confirmed by the expression of luciferase in both xenograft models by Xenogen IVIS Kinetic imaging system (Figure 4C, left panel).

Before *in vivo* study, gy1 was labeled with a near infrared dye, IRDye800CW-NHS ester, and binding ability of the labeled gy1 was confirmed by flow cytometry after incubation with PC3-PSMA^+^ cells (Figure 4B). IRDye800CW labeled gy1 was then injected into PSMA positive and negative PC3 xenograft nude mice through tail vein, and the distribution of gy1 was monitored using near-infrared fluorescence imaging (FLI) at different time points. Results showed that the IRDye800CW labeled gy1 diffused rapidly throughout the whole body after 1 h and can be detected in tumor tissues from 2 h. While IRDye800CW labeled gy1 was then gradually cleared from the body later on, specifically retainment was observed in PSMA positive, but not negative tumor tissues. Highest signal/background ratio in tumor was obtained at 6 h post-injection, and the signal in tumor was nearly undetectable after 24 h (Figure 4C, right panel, Supplementary Figure 3).

Mice were sacrificed at 12 h after gy1 injection and different tissues were collected for further bio-distribution evaluation. In the PC3-PSMA^+^ group, consistent with the FLI data, strongest fluorescent signal can be detected in tumor tissues, weak signals can be detected in kidney, liver and spleen, while only negligible signals can be detected in other tissues. While in the PC3-PSMA^−^ group, no obvious fluorescent signal can be detected in tumor tissues (Figure 4D, 4E). These data suggested that gy1 can specifically target PSMA positive tumor *in vivo*, which encourage the development of PSMA targeted imaging and therapy using gy1, such as intraoperative optical imaging, PET imaging, nanomedicine and antibody drug conjugate.

## DISCUSSION

Antibody is the most efficient and accurate tool for tumor targeting, which can not only be applied in tumor diagnosis, but also can help surgeons to clearly find out the tumor margin, confirm invasion and metastases in a real-time manner [14, 15]. PSMA is a type II membrane protein, which has been identified to be specifically expressed in prostate cancer and tumor vasculature, providing an ideal target for tumor imaging and therapy.

So far only one PSMA-targeting antibody has been approved by FDA for diagnosis, which is 7E11-C5 conjugated with ^111^In (^111^In-capromab pendetide, Prostascint®) for nuclear imaging. However, this antibody has not been widely used because it binds to the intracellular domain of PSMA and therefore can only access to its target in damaged or necrotic cancer cells [16, 17]. There do are some other antibodies, such as J591, D2B, and 3/A12, that target the extracellular domain of PSMA and have been widely used in research and clinical trials [18–22]. However, the fact that their mouse origin may cause significant side effects when used in human greatly restrains their further application in clinic. Furthermore, their affinity is not high enough, for example, the affinity of J591 full antibody is only 3 nM, while the scFv derived from it is even lower [20, 23]. The antibodies developed by PSMA Development Company, LLC using xeno-mouse are very impressive since they are fully human origin and have high binding affinity [24, 25]. Nevertheless, an appropriate clearance rates is very important for targeted imaging, especially for nuclear imaging because unnecessary prolonged exposure to radiation may cause carcinogenesis. Full antibody based imaging usually need about one week to obtain a good signal/background ratio [18, 26], while smaller antibodies can reach the best signal/background ratio within 24 h, thus are more favorable for clinical imaging.

ScFvs have simple structure that allows lower cost production using *E.coli*, and the small size of scFvs enables deep penetration into tumor and faster clearance from circulation. Thus, scFv is ideal for targeted imaging. Overall, as long as the affinity is high enough, scFvs possess unique advantages over full antibodies in tumor targeted imaging and therapy. Full antibodies could be converted into scFvs. However, after conversion, the affinity usually drops more than ten times and even unpredictably lower due to the structure change and the transition between dimmer to monomer.

Yeast display antibody library provides a great source to obtain novel antibodies because it allows eukaryotic expression and direct isolation of antibodies via fluorescence activated cell sorting. However, the low transformation efficiency of yeast strains makes it difficult to construct large antibody libraries, which, as a result, severely limited its application for novel antibody discovery. We have overcome this obstacle and constructed a 1×10^11^ naive human scFv library from peripheral blood mononuclear cells that were isolated from 5 liter peripheral blood from 56 healthy donors [11]. PSMA has a big extracellular domain, which provides great chance to isolate high affinity antibodies targeting PSMA from antibody libraries [27]. We panned the large scFv library using the extracellular domain of PSMA, and obtained a high affinity anti-PSMA scFv gy1. To evaluate the potential of gy1 for molecular imaging and therapy for PCa, we expressed gy1 in prokaryotic expression system and evaluate its bioactivity both *in vitro* and *in vivo*.

Our result showed that the binding affinity of *E. coli* expressed gy1 is pretty high, and gy1 can rapidly and significantly internalize into PSMA positive cancer cells through endosome-lysosome pathway. This character enables gy1 to be applied for the development of antibody drug conjugate (ADC) and nanoparticles for specific tumor killing [28, 29]. The low pH and high level of cathepsins in lysosome are favorable for cleavage of pH sensitive linkers for ADC or nanoparticles to efficiently release payload [30–32]. Therefore, the high binding affinity and excellent internalization properties also make gy1 and its derived antibodies ideal vehicles for therapeutic drug delivery.

For optical tumor imaging, Near infrared (NIR) dyes are favorable due to their deep penetration in tissues. Indocyanine green (ICG) is the only FDA approved NIR dye and has been used for full antibody labeling [33, 34]. But its hydrophobic property limits its application in scFv labeling because scFv is not well tolerant to organic solvents. IRDye800CW is another safe NIR dye for labeling, which is highly hydrophilic and is convenient for scFv labeling in PBS buffer [35]. Furthermore, except metabolism in liver, this dye does not target elsewhere *in vivo*. In this study, we labeled gy1 with IRDye800CW and demonstrated the highly efficient tumor targeting of gy1 in PSMA^+^ tumor tissues *in vivo*. Gy1 is a fully human scFv and IRDye800CW is a safe dye, their combination is therefore expected to be a safe and highly efficient agent for PCa diagnostic and/or intraoperative imaging.

In summary, we have successfully isolated a high affinity fully human anti-PSMA scFv (named gy1) from a large human naive scFv library. We have demonstrated its application for *in vivo* optical imaging for PCa when labeled with NIR dye IRDye800CW. The binding of gy1 with PSMA can induce rapid and significant internalization into PSMA positive cells through the endosome-lysosome pathway, indicating that gy1 is not only a great probe for molecular imaging, but also can be used as an excellent delivery vehicle for toxic drugs for PCa therapy.

## MATERIALS AND METHODS

### Cell culture

Human prostate cancer LNCaP C4-2 and PC3 cells were maintained in RPMI 1640 or F12K medium(Gibco Life technologies, Paisley, Scotland, UK) supplemented with 10% fetal bovine serum (Gibco Life technologies, Paisley, Scotland, UK) and 1% penicillin-streptomycin (Invitrogen Life technologies, Carlsbad, CA, USA). PC3 cells were infected with firefly luciferase expressing lentivirus and PSMA expressing lentivirus or control lentivirus and selected by puromycin. The stable PSMA and luciferase expressing PC3 cells and luciferase expressing control cells were named PC3-PSMA^+^ and PC3-PSMA^−^ cells respectively. All the cell lines were cultured at 37^°^C with 5% CO_2_ in a humidified incubator.

### Panning of a yeast display scFv library

A 1×10^11^ naive yeast display scFv library (OriMAbs Ltd, PA, USA) was used for panning to isolate anti-PSMA scFvs using extracellular domain recombinant human PSMA protein (R&D systems). The PSMA protein was labeled with biotin before being used for panning. The library was panned at a progressively decreasing concentration (from 40 μg/200 ml to 1 μg/3 ml) and the antigen binding yeast population was sorted using streptavidin or anti-biotin antibody conjugated microbeads (Miltenyi Biotec, Bergisch Gladbach, Germany) alternatively. Magnetic sorting was followed by three rounds of flow sorting to further enrich high affinity yeast clones. The top 0.1% population was sorted during the last sorting, and the yeast plasmid was isolated after expansion. The scFv gene fragments were amplified by PCR using the following primers, Forward: 5′-GACTACAAGGACGACGATGAC-3′, and Reverse: 5′-AGTAGAATCAAGACCTAGTAGAGGG-3′. The amplified scFv gene fragments were then purified and co-transformed into yeast strain YVH10 along with Sfi I /Not I linearized secretory scFv expression vector pYS1 (OriMAbs Ltd, PA, USA). The molar ratio of the scFv gene/vector was 3:1 and 1μg vector was used for transformation. Three hundred and eighty four clones were picked, cultured and secretory scFv expression was induced for 2 days at 20^°^C as described [11].

### High throughput ELISA to identify PSMA specific scFvs

PSMA specific scFv clones were identified using high throughput ELISA. The ELISA plates were coated with anti-Flag antibody (Sigma, St. Louis, MO, USA) overnight at 4^°^C, blocked with PBST (PBS containing 0.05% Tween 20) containing 5% nonfat dry milk (PBSTM) (Bio-rad, Valencia, CA), and then incubated with the scFv containing yeast supernatant diluted with equal volume of PBSTM. Then 0.4 μg/ml biotinylated PSMA was added into the plates and the captured PSMA was detected by Streptavidin-HRP (BD Biosciences, CA, USA) and colorimetric signals were developed with TMB substrate solution (KPL, Inc., Gaithersburg, MD), quenched with sulfuric acid (KPL, Inc) and the absorption was read at 450 nm. Ninety six positive clones were selected for further identification using recombinant Fc protein as a negative control. Clones only reacting to PSMA, but not Fc fragment, were sent for sequencing to get the genes of the scFv fragments.

### Gy1 expression in yeast and affinity measurement

A high scFv expressing YVH10 clone was expanded and induced for scFv protein production for 4 days at 20^°^C. A 6His tag was designed at the C terminal of scFv to facilitate the purification of the scFv from the yeast supernatant using Nickel resin. The affinity of the scFv was measured using ELISA described above except that the scFv concentrations were diluted in three fold serial dilutions from 100 nM down to 0.137 nM. The affinity was calculated using GraphPad Prism software.

### Gy1 expression and purification in *E.coli*

The gy1 gene was amplified and cloned into the prokaryotic expression vector pET302 (named pET302-gy1), then transformed into *E. coli* BL21 and induced to expression by 0.05 mM isopropyl-1-thio-b-galactopyranoside (IPTG) for 4 h at 30^°^C. The gy1 protein was then purified by affinity chromatography using Ni^2+^-NTA column (Qiagen, Valencia, CA, USA) according to the manufacturer's protocol. An anti-HER2 scFv (named NCP1) was expressed and purified in the same way and was used as negative control.

### SDS-PAGE and western blot

For SDS-PAGE analysis, protein samples were separated by 12% SDS-PAGE and the gel was stained with Coomassie blue and de-stained with destaining buffer. For western blot analysis, the SDS-PAGE separated proteins were transferred onto polyvinylidene difluoride (PVDF) membranes. The membranes were then blocked with 5% nonfat milk diluted in PBS for 2 h at room temperature before being incubated with anti-6His primary antibody (1:1000; Cell Signaling Technology, Danvers, MA, USA) overnight at 4^°^C. Membranes were then washed and incubated with HRP-conjugated Goat anti-Mouse IgG (1:5000; Genshare Biological, Xi'an, China) for 1 h at room temperature, and the antibody-bound proteins were visualized using a MultiImage™ Light Cabinet Filter Positions (Alpha Innotech Corporation, San Leandro, CA, USA).

### Cellular ELISA

To evaluate the binding affinity of gy1 to membrane expressed PSMA, PSMA positive C4-2 cells were seeded at 5 × 10^4^ per well in 96-well plate and cultured overnight. The second day, cells were fixed with 4% paraformaldehyde for 20 min before being treated with 3% H_2_O_2_ for 20 min to block endogenous peroxidase followed by blocking with 6% bovine serum albumin for 30 min at room temperature. Three-fold serially diluted gy1 and the control scFv NCP1, from 8100 nM down to 0.005 nM, were added and incubated for 1 h at 37^°^C. Cells were then washed with PBST and incubated with HRP-conjugated mouse anti-6His antibody (AbD Serotec, Bio-Rad, Oxford, UK) for 1 h at room temperature. Colorimetric signals were developed by addition of 3, 3′, 5, 5′-tetramethylbenzidine (TMB, eBioscience, CA, USA) and stopped by incubation with 1 M H_2_SO_4_ for 15 min. The absorbance was measured at 450 nm using a Sunrise microplate reader (Tecan, Groedig, Austria) and the binding curves were analyzed using GraphPad Prism 5.0 software. The gy1 affinity was calculated using non-linear regression analysis of a one-site binding hyperbola equation. Kd value was presented from three independent experiments, and each independent experiment was performed in quadruplicate.

### Flow cytometry

Prostate cancer cells, LNCaP, PC3, C4-2, PC3-PSMA^+^ and PC3-PSMA^−^ cells were detached with Versene solution (1.37 M NaCl, 26.8 mMKCl, 80.7 mM Na_2_HPO_4_, 14.7 mM KH_2_PO4, 5.4 mM disodium EDTA, 0.2% D-glucose) and suspended in PBS at a density of 1×10^6^ cells/mL, cells were washed with FACS buffer (PBS containing 0.2% bovine serum albumin and 0.05% sodium azide) before staining. Cells were then incubated with PE conjugated anti-PSMA IgG (Biolegend, CA, USA) for 30 min at 4^°^C in darkness. After being washed with FACS buffer, cells were analyzed by flow cytometry (BD Bioscience). To evaluate the binding capability of gy1 to PSMA expressing cancer cells, cell suspension were incubated with 100 nM gy1 or control scFv NCP1 at 4^°^C for 30 min, followed by washing and incubation with FITC-conjugated mouse anti-6His IgG (AbD Serotec; Bio-Rad) for 30 min at 4^°^C in darkness. Cells were then washed and analyzed by flow cytometry.

### Immunofluorescent staining

Cells grown on coverslips at 50% confluence were incubated with 200 nM gy1 or NCP1 for 2 h at 37^°^C. Cells were washed, fixed with 4% paraformaldehyde for 20 min. Internalized gy1 was detected by FITC-conjugated mouse anti-6His IgG (AbD Serotec; Bio-Rad). Cell were then stained with 4′,6-diamidino-2-phenylindole (DAPI) to visualize the nuclei. Finally cells were washed with PBS and mounted on slides. Slides were observed and pictured under laser scanning confocal microscopy (FluoView FV1000, Olympus).

Staining of the cellular organelles were performed using CellLight® Reagents (Invitrogen Life technologies, CA, USA) including Lysosomes-RFP, Endosomes-RFP, Golgi-RFP and ER-RFP according to the manufacturer's protocol. Cell images were captured by laser scanning confocal microscopy (FluoView FV1000, Olympus).

### Animal model and *in vivo* optical imaging study

BALB/c nude mice (4-6 weeks old, male, body weight 20-30 g) were purchased from the animal center in Fourth Military Medical University and maintained under specific pathogen-free conditions. All the animal work was performed according to the protocol approved by the Guidelines for the Care and Use of Laboratory Animals of Fourth Military Medical University. The xenograft tumor models were developed by injecting 5×10^6^ firefly luciferase-expressing PC3-PSMA^+^ or PC3-PSMA^−^ cells in 0.2 mL PBS subcutaneously in the right flank of each mouse.

For *in vivo* optical imaging study, the gy1 was labeled with IRDye800CW using IRDye800CW labeling kit (Li-Cor Biosciences, Nebraska, USA). Extra dye was removed using dialysis bag, with the molecular-weight cutoff ≥10K. Dialysis was maintained for 8 hours, during which PBS buffer was changed three times. The labeled antibody was then concentrated using ultrafiltration tube. For each mouse, 0.2 μmol/kg of the IRDye800CW-labeled gy1 was injected intravenously, and the mouse was anesthetized at indicated time point and the IRDye800CW fluorescence was monitored under the Xenogen IVIS Kinetic imaging system at an excitation wavelength of 745 nm. Identical illumination settings (1 second exposure, f/stop = 2) were used for all images. In parallel with the near-infrared fluorescence imaging (FLI), five mice in each group treated for 12 h were sacrificed and different tissues were isolated and fluorescence intensities were analyzed. Fluorescence intensities were calculated using Living Image software and presented as photon flux (p/s/cm2/sr).

### Immunohistochemistry

Histologic slides were prepared from formalin-fixed, paraffin-embedded animal tissues and sectioned at 5 μm thickness. Tissue slides were deparaffinized in xylene and rehydrated through a graded alcohol series. The slides were then placed in 10 mM citrate buffer (pH 6.0) and microwaved at 900 W for 20 min to achieve antigen retrieval. Slides were treated with 3% H_2_O_2_ for 10 min to block endogenous peroxidase followed by incubation with 5% bovine serum albumin for 30 min at room temperature to block nonspecific binding. Sides were then incubated with anti-PSMA antibody (1:100, Cell signaling technology) at 4^°^C overnight, then washed and incubated with HRP-conjugated Goat anti-Rabbit IgG (Dako, ChemMate, Danmark) for 30 min at room temperature. PSMA expression was visualized using 3,3′-diaminobenzidine (DAB) chromogen staining for 2-3 min.

## SUPPLEMENTARY FIGURES


