# Supplementary material for: A novel anti-PSMA human scFv has the potential to be used as a diagnostic tool in prostate cancer
Source: Oncotarget. 2016 Jul 19;7(37):59471–81. doi: 10.18632/oncotarget.10697 (PMC5312325; doi:10.18632/oncotarget.10697)
Supplement: Supplementary file 1 [file oncotarget-07-59471-s001.pdf]

A novel anti-PSMA human scFv has the potential to be used as a diagnostic tool in prostate cancer

SUPPLEMENTARY FIGURES

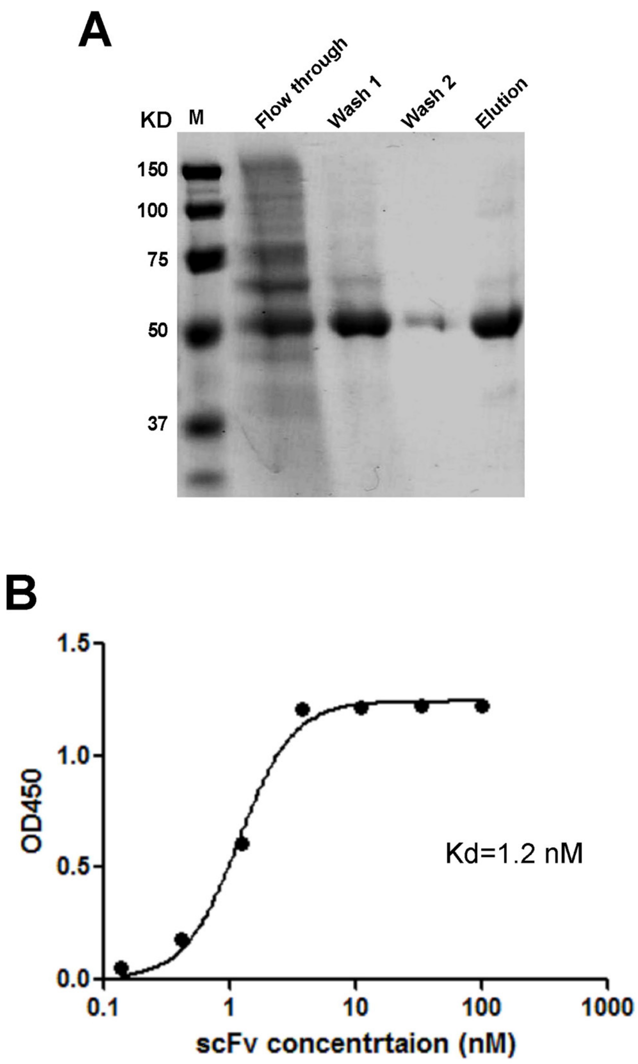

Supplementary Figure S1: Expression, purification of gy1 in yeast and affinity measurement by capture ELISA. A. Purification of yeast expressed gy1 scFv. **B.** Affinity measurement of gy1 by capture ELISA. The Kd was calculated using non-linear regression analysis of a one-site binding hyperbola equation by GraphPad Prism 5.0 software. Representative result was shown from 3 independent experiments.

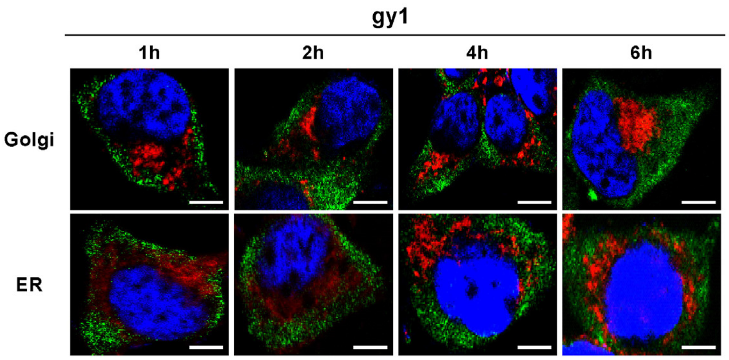

Supplementary Figure S2: Gy1 is not co-localized with Golgi or ER marker at different time points. Immunofluorescent staining to examine the potential co-localization of gy1 with Golgi or ER marker at different time points. C4-2 cells were incubated with 200 nM gy1 for different period of time and then stained by anti-6His IgG and FITC-conjugated secondary antibody. Golgi or ER marker (RFP-labeled) and nucleus (blue) were co-stained. Scale bar = 10 μm

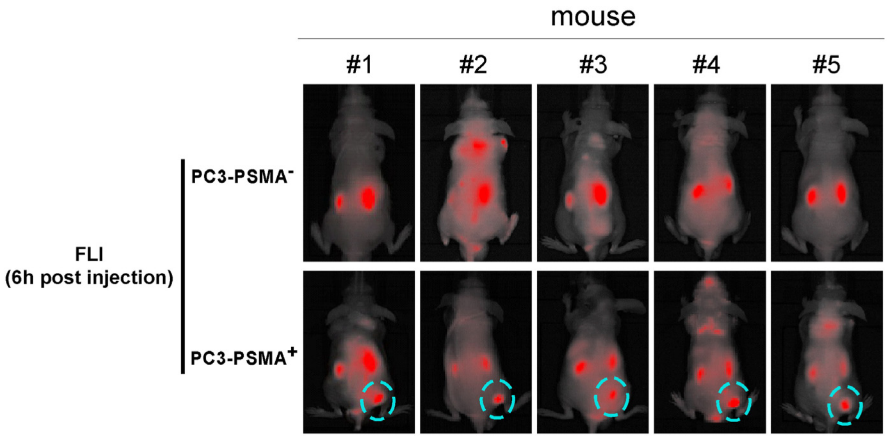

Supplementary Figure S3: Bio-distribution of IRDye800CW-labeled gy1 in five pairs of PC3-PSMA$^+$ or PC3-PSMA$^-$ xenograft nude mice. The localization of IRDye800CW-labeled gy1 was monitored at 6 h by fluorescence imaging excited at 745 nm after intravenous injection.
